# The multiform sonographic spectrum of arterial duct in right aortic arch

**DOI:** 10.1007/s10554-021-02325-w

**Published:** 2021-07-08

**Authors:** Enrico Chiappa, Cecilia Ridolfi, Adalgisa Cordisco

**Affiliations:** 1 Division of Pediatric Cardiology, National Research Center—Tuscany Region Foundation “G. Monasterio”, Ospedale del Cuore, via Aurelia Sud, 54100 Massa, Italy; 2 Specialization School in Diseases of Cardiovascular System, University of Pisa, AOU Pisana, via Paradisa 2, 56124 Pisa, Italy; 3 Division of Prenatal Diagnosis Center, P. Palagi Hospital, viale Michelangiolo 41, 50141 Florence, Italy

**Keywords:** Arterial duct, Fetus, Prenatal diagnosis, Right aortic arch, Vascular ring

## Abstract

To study the different characteristics of arterial duct (AD) in a series of prenatally detected right aortic arch (RAA). Out of 832 congenital heart diseases (CHD) referred to a tertiary center, 98 cases had RAA. Based on anatomical landmarks we identified 7 types of AD: *type 1* left-sided, transverse; *type 2* left-sided, vertical; *type 3* from the underside of aortic arch (AA), vertical; *type 4* right-sided, mirror-image “V”, transverse; *type 5* right-sided, “H” shaped, transverse; *type 6* bilateral; *type 7* absent or unidentifiable. For each type of AD the incidence of associated major CHD was calculated and chi-square test was applied to verify the null hypothesis with significance level of p < 0.05. Type 1 occurred in 43% of cases including 4 with CHD and no cases with pulmonary outflow obstruction (POO). Symptoms of vascular ring were present in 41% of survivors. Type 2, 3 and 7 AD were associated with tetralogy of Fallot (TOF) or equivalents. No type 5 AD with CHD had POO and 3 isolated cases had asymptomatic hypoplasia of left pulmonary artery (LPA). Two type 6 AD had disconnection of LPA. Type 1 occurred more often as an isolated finding (p < 0.001), whereas types 2 (p = 0.0026), 3 (p = 0.0045), 4 (p = 0.0325) and 7 (p = 0.0001) were frequently associated with major CHD. In RAA, type 1 (U-shaped) is usually an isolated finding (p < 0.001) which includes all symptomatic vascular rings. POO is always present when the AD is vertical or absent but not when it lies on a transverse plane. Bilateral AD is rare and brings the risk of functionary loss of left lung if not identified.

**Supplementary Information:**

The online version contains supplementary material available at 10.1007/s10554-021-02325-w.

## Introduction

In the embryo, the arterial duct (AD) normally originates from the sixth branchial arch between weeks 6–8 of gestation [1]. Unlike the right side, the terminal tract of the left-sided sixth branchial arch does not regress and becomes the AD linking the aorta and the pulmonary trunk. This is an important shunt in the fetal circulation, which allows blood from the right ventricle to bypass the pulmonary circulation and enter the systemic circulation.

AD and aortic arch (AA) anomalies are now easily assessed in prenatal evaluation with ultrasound. These vessels can be visualized with fetal thorax scan plans, which cannot be obtained postnatally due to lung ventilation. The AD can only be studied within hours postnatally, whilst still patent. After functional closure and transformation into the arterial ligament, the origin and course of the AD is no longer visible with any current techniques, and can only be deduced by displaying the ductal ampulla on the aortic side and/or the ductal diverticulum on the pulmonary artery side.

Variants of AA anomalies are characterized by laterality and branching pattern, and the location and course of the AD. Prevalence of right aortic arch (RAA) in adults is estimated at around 0.1% with higher incidence in prenatal low-risk populations [2]. Excluding cases with aberrant right subclavian artery in left-sided AA, most AA anomalies have one common sonographic marker in prenatal diagnosis: the AA (or one of the arches) is visualized on the right side of the trachea in the axial views of the upper mediastinum. The three-vessel and trachea view is becoming a routine assessment in fetal scans, complementary to the four-chamber view [3–6]. RAA is therefore being detected increasingly frequently. Most recent papers published on RAA have focused on the modality of bifurcation of the head and neck vessels disregarding often the characteristics of the AD [7–12].

We aimed to retrospectively revise all RAA cases referred to our fetal cardiology unit, analyze and classify the different characteristics of AD, and define an ultrasound method for their identification. We also analyzed postnatal data in cases not associated with congenital heart diseases (CHD) for symptoms or complications.

## Methods

### Subjects

Out of 832 cases of CHD referred to our tertiary fetal cardiology unit between January 2005 and December 2019, 98 consecutive cases of RAA were identified. Data from the medical records of the mother and child were systematically reviewed. Prenatal diagnosis of RAA was verified in all patients by transthoracic echocardiography, surgery reports, or autopsy findings. Postnatal MRI, CT or bronchoscopy were performed in selected patients. The need for ethical approval was waived due to the retrospective design of the study.

### Echocardiography

Fetal and postnatal echocardiography was carried out using a Philips IE 33 or a Philips Epiq 7G ultrasound machine with 8–3 and/or 5–2-MHz sector transducers (Philips, Andover, MA, USA). Examinations were carried out in a segmental approach using standardized anatomical views with special attention to the basic transverse views of the fetal thorax. RAA was defined as when the transverse AA passed to the right of the trachea. The definition of the AD was based on site of origin, alignment to major body planes, and relationship with the trachea. Based on these parameters we identified seven different types of AD described in the following sections.

### Type 1—left-sided, transverse

**Fig. 1:**
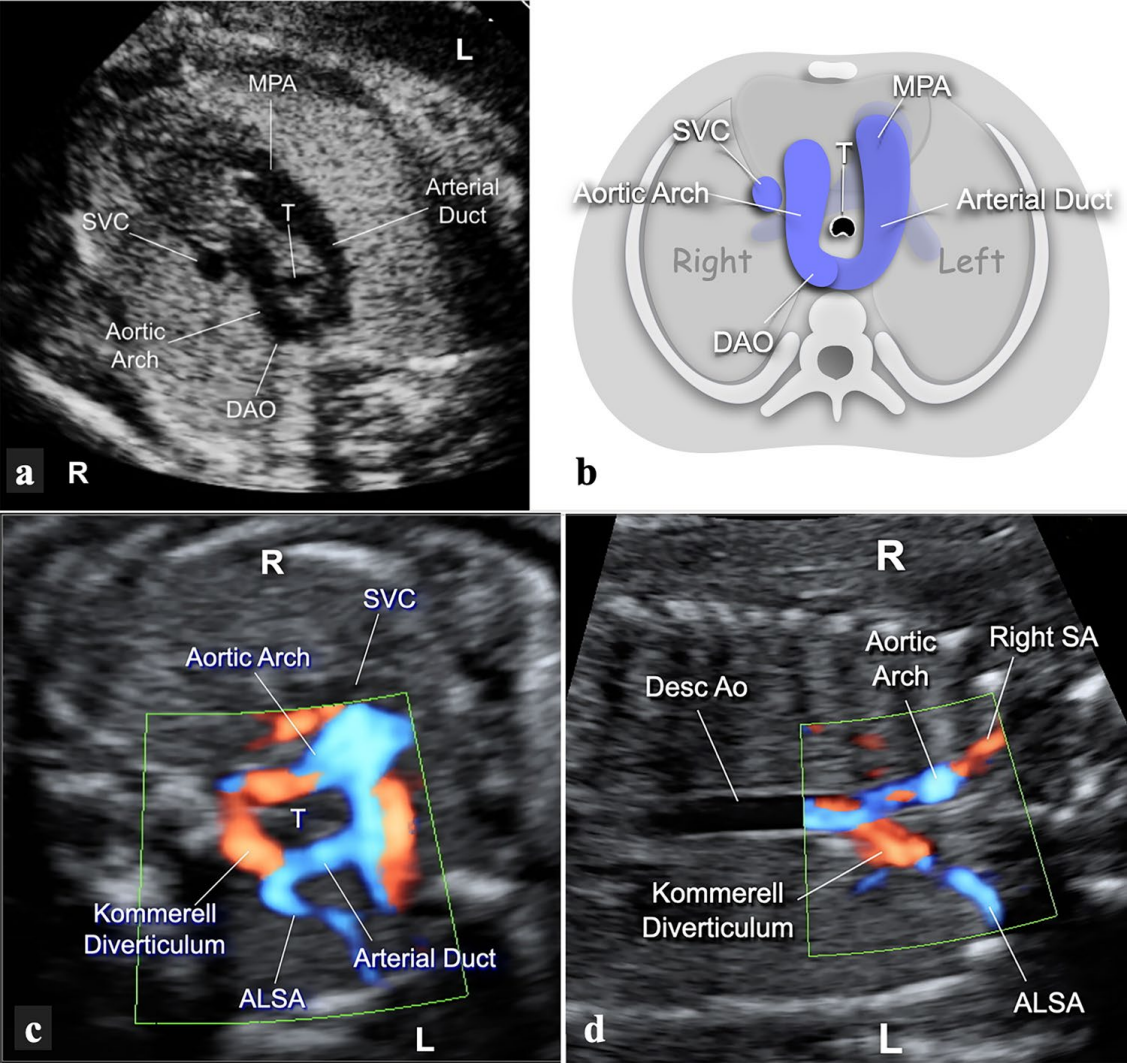
**a**, **b** Gray-scale and the corresponding cartoon of the 3-vessel and trachea view in a fetus with RAA and and left AD. The transverse AA lies to the right and the AD to the left of the trachea. The shape of the two arches resembles that of the letter “U”. With sections at this level, it is not possible to distinguish the different pattern of branching of the head and neck vessels. **c** Transverse view of the upper mediastinum in a 22-week gestational-age fetus with RAA, left AD and ALSA. The fetal thorax is approached from the right side to obtain a more parallel alignment with the ALSA. The AA and the AD are seen on opposite sides of the trachea, and merge together posteriorly to the trachea, through the Kommerell’s diverticulum. The origin of the ALSA is clearly shown. **d** Coronal view of the thorax in the same case. The use of power-Doppler allows better highlighting of the flow inside the vessels despite the unfavorable alignment with the ultrasound beam. In the upper part of the picture, the right-sided aorta is seen joining the Kommerell’s diverticulum (red color) to form the descending aorta in a “Y”-shaped fashion. The ALSA is shown in the inferior part of the picture. *ALSA* aberrant left subclavian artery, *DAO* descending aorta, *L* left, *MPA* main pulmonary artery, *R* right, *SVC* superior vena cava, *T* trachea

**Fig. 2:**
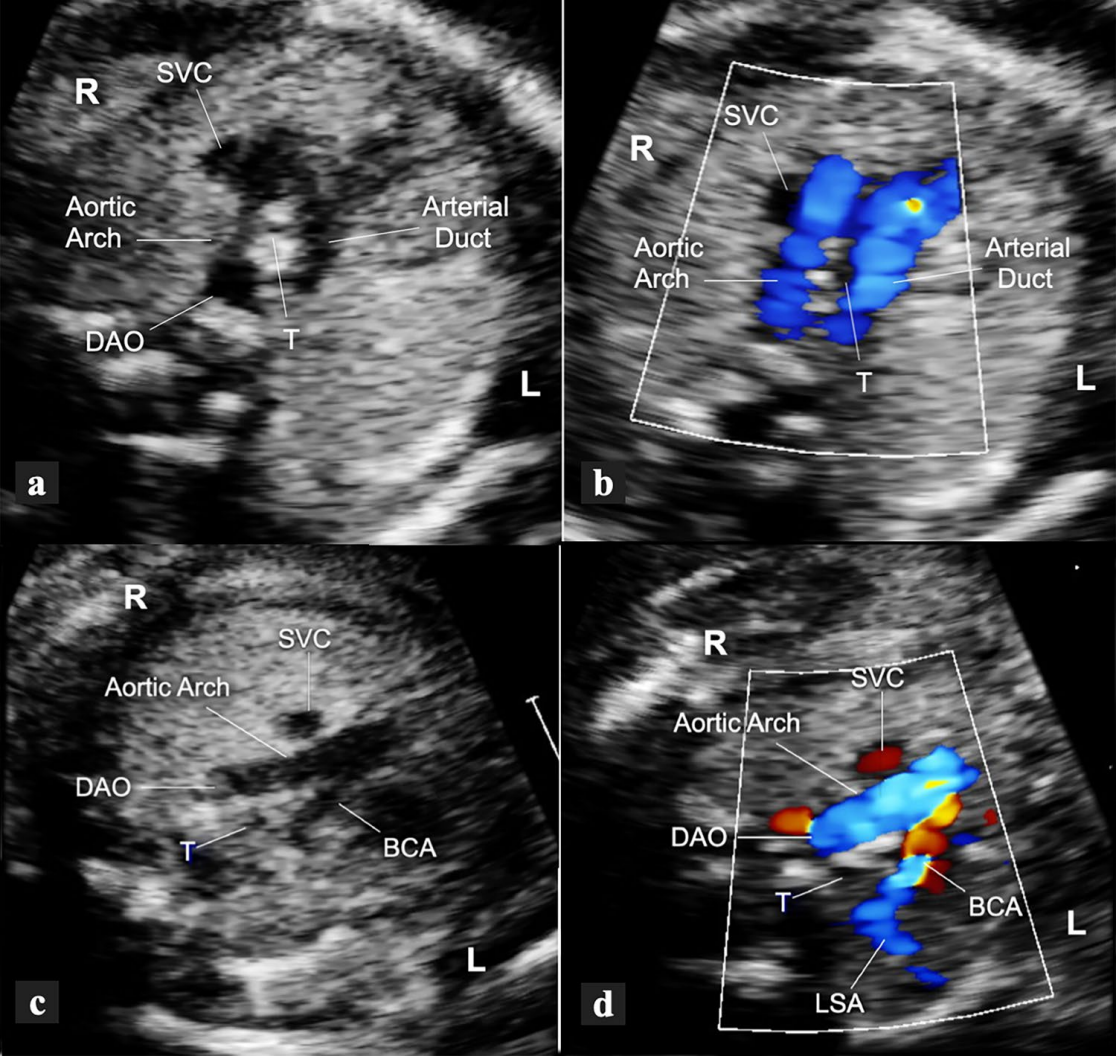
Transverse view of the upper mediastinum in a 22-week gestational-age fetus with RAA, left AD and mirror-image branching (type 1b AD). The aortic arch is seen on the right side of the trachea while the AD is on the left, thus forming a “U”-shaped vascular loop around the trachea (**a**, **b**). With slight cranial tilt of the scanning plane (**c**, **d**), the left-sided brachiocephalic artery is seen, giving origin to the left subclavian artery distally. *BCA* brachiocephalic artery, *DAO* descending aorta, *L* left, *LSA* left subclavian artery, *R* right, *SVC* superior vena cava, *T* trachea

**Fig. 3:**
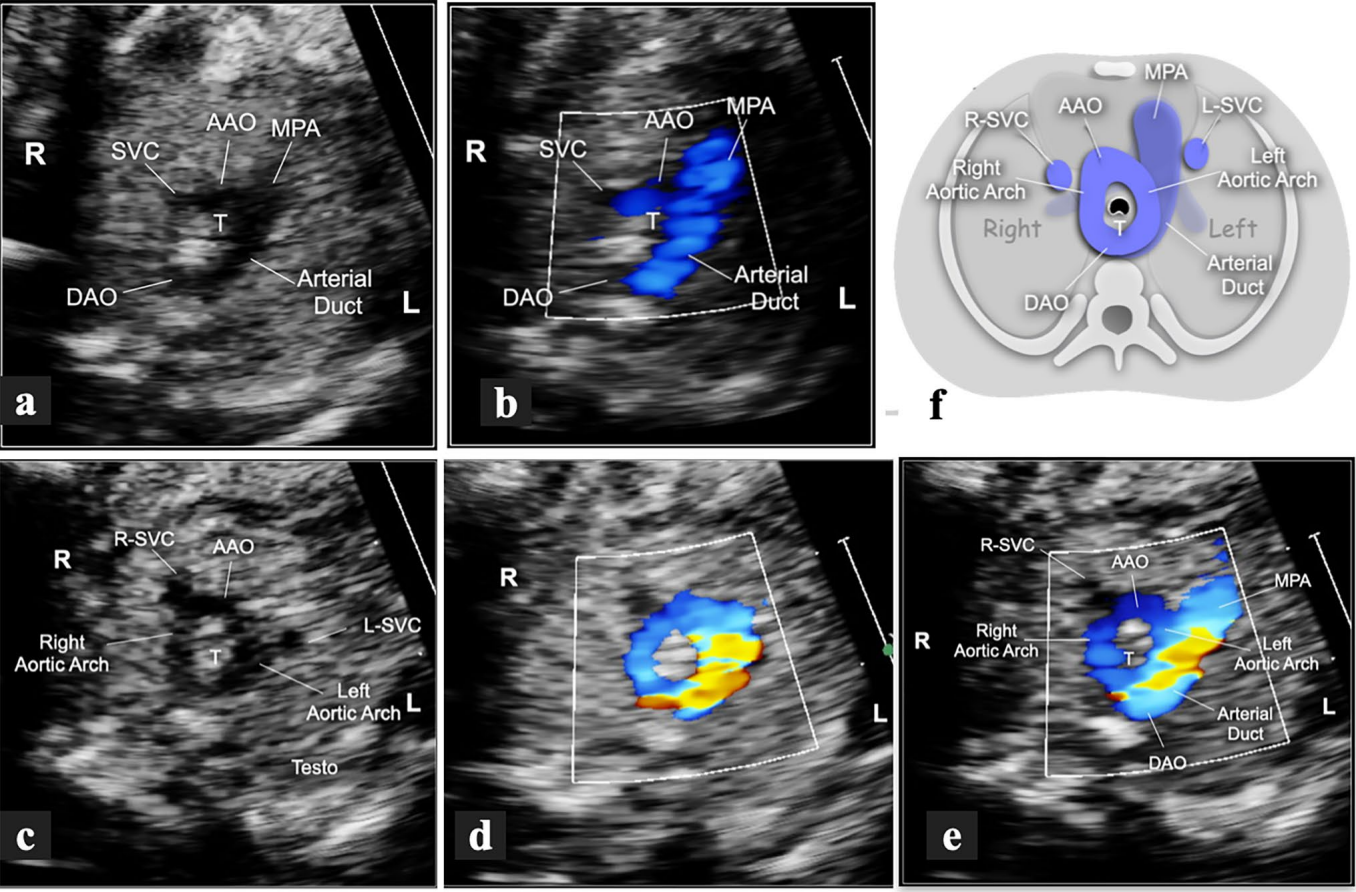
Short-axis views of a fetus at 22 weeks with double aortic arch and left-sided arterial duct. The three-vessel view in gray-scale (**a**) and CFM (**b**) shows a left-sided arterial duct running from the main pulmonary artery to join the descending aorta, behind the trachea, on the right-anterior corner of the spine. The position of the descending aorta anticipates a right-sided aortic arch. Transverse sections with slightly cranial angulation in the same fetus (**c**-**e**). These views show the two aortic arches originating from the ascending aorta, embracing the trachea on both sides and merging posteriorly into the descending aorta. In addition to a normal right-sided superior vena cava, a persistent left superior vena cava was associated. If only the innermost portion of the vascular ring is included, the image will resemble a letter “O” (**c**, **d**). With slight caudal orientation, the shapes of the two arches with the main pulmonary artery resemble that of a letter “d”. Schematic representation of the same vascular anomalies (**f**). This cartoon highlights the relationship between the two aortic arches, the trachea and the arterial duct underneath, represented in dark blue. *AAO* ascending aorta, *DAO* descending aorta, *L* left, *MPA* main pulmonary artery, *R* right, *SVC* superior vena cava, *T* trachea

The AD lies on the transverse plane on the left side of the trachea, extending from the proximal left pulmonary artery (LPA) to the descending aorta (Fig. 1a, b). In this group, we further distinguished three different branching patterns of the head and neck vessels:

- (a) With aberrant left subclavian artery (ALSA). To identify these cases, a transverse view of the upper mediastinum with slight cranial angulation was employed to visualize the origin of the ALSA (Fig. 1c). When the fetal position allowed it, a coronal view of the fetal thorax was further employed. This view allows visualization of a long stretch of the descending aorta ventral to the spine, and the confluence between the AA and the AD where the Kommerell’s diverticulum and the origin of the ALSA are located (Fig. 1d).
- (b) With mirror-image branching. A transverse view of the upper mediastinum slightly cranial to the plane of the AA and AD will show the brachiocephalic artery originating from the proximal AA, angled to the left, and giving origin to the left subclavian artery (Fig. 2).
- (c) With double aortic arch (DAA). In the large majority of these cases, the AD is left-sided (Fig. 3a, b). Depending on the scanning plane inclination and image orientation, the vascular ring can be visualized in slightly different ways, shaped like the number “6”, a lowercase “d”, or an uppercase “O” (Fig. 3c–e).

### Type 2—left-sided, vertical

**Fig. 4:**
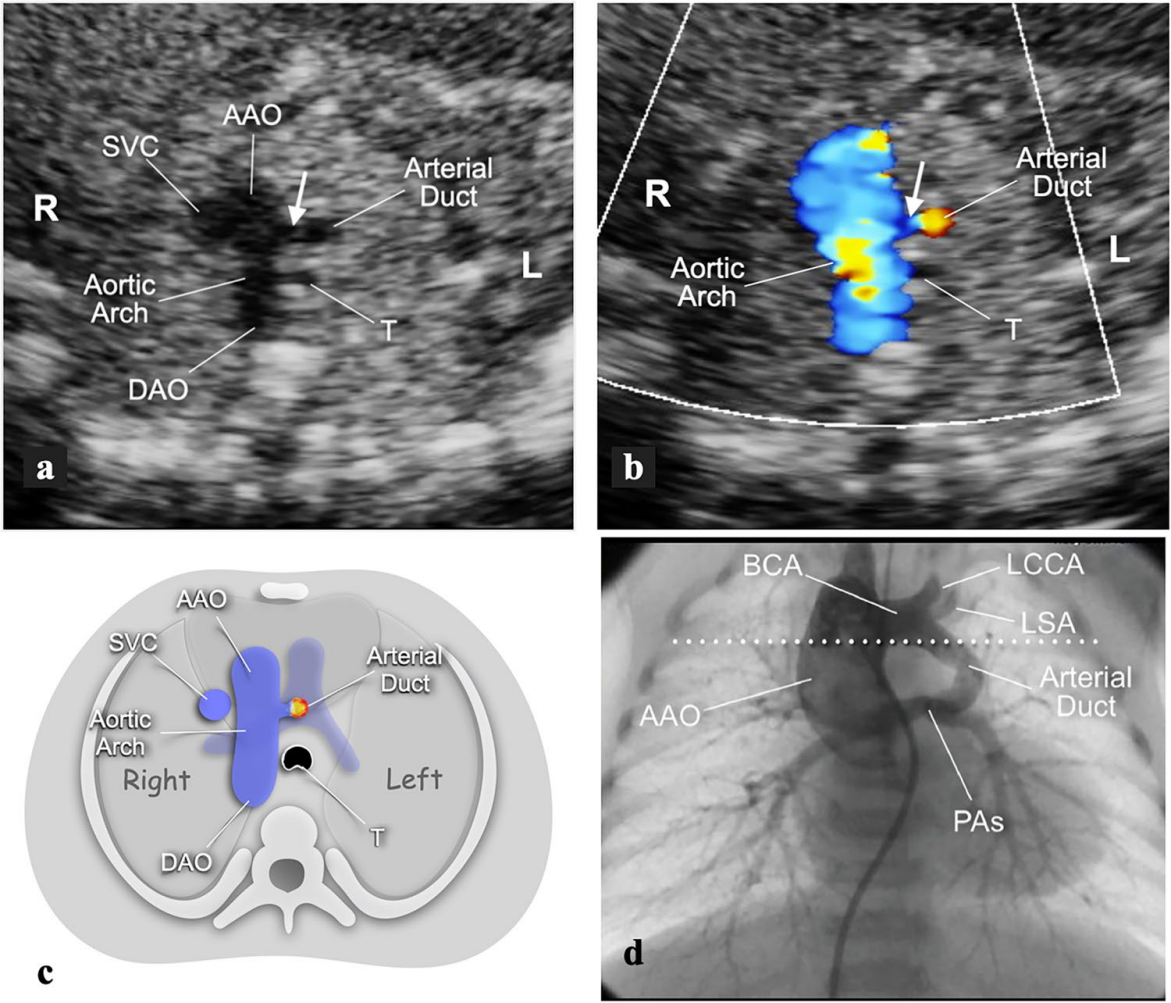
Transverse views of the upper mediastinum in a 20-week gestational-age fetus with RAA and type 2 AD. The gray-scale image (**a**) shows the transverse AA to the right of the trachea. To the left of the proximal AA, a cross section of the AD is seen. The white arrow indicates the lower portion of the brachiocephalic artery from which the AD originates. With color flow mapping and a low scale setting (**b**), the AD is color-coded despite the unfavorable alignment with the ultrasound beam. The cartoon (**c**) allows better appreciation of the relationships between the AD and the confluence of the pulmonary arteries underneath, in light blue. This example was associated with TOF and severe obstruction to the pulmonary outflow. **d** Neonatal aortography, in anterior–posterior projection, in the same case as in a and b. This image shows the left-sided brachiocephalic artery with RAA. From the lower portion of this artery, the AD runs on a vertical plane and with slightly arched course to join the confluence of the pulmonary arteries inferiorly. The white dotted line indicates the level of section planes shown in Fig. 4a, b. *AAO* ascending aorta, *BCA* brachiocephalic artery, *DAO* descending aorta, *L* left, *LCCA* left common carotid artery, *LSA* left subclavian artery, *PAs* confluence of pulmonary arteries, *R* right, *SVC* superior vena cava, *T* trachea

This type of AD is associated with mirror-image branching of the head and neck vessels, and there is therefore no aberrant subclavian artery. The AD runs with a vertical course between the root of the left-sided subclavian artery and the left PA below, and leaves the trachea posteriorly. Therefore, no vascular ring is formed. Although difficult, this type of AD can be identified in transverse views of the fetal upper mediastinum. In these sections, the transverse AA has an appearance like a “sausage”, with antero-posterior orientation. A transverse section of the AD will be seen to the left of the AA, joined by a short vascular isthmus, which is the lower portion of the brachiocephalic artery. This view resembles the characters “I-o”, where “I” refers to the AA, the lowercase “o” to the transverse section of the AD, and the conjoining “dash” to the inferior portion of the left brachiocephalic artery (Fig. 4).

### Type 3—from the underside of the AA, vertical

This type of AD lies on a vertical plane and runs from the inferior portion of the AA to the underlying bifurcation of the PA or one of its branches. In axial views of the upper mediastinum, the AD will be seen in cross section between the oblique sections of the proximal and distal AA (Suppl. Fig. Ia-c). A long-axis view of the AA will confirm this pattern (Suppl. Fig. Id, e). Usually, the AD is small with retrograde flow from the AA to the pulmonary arteries. This type is not exclusive to right-sided AA, but characteristic of CHD with duct-dependent pulmonary circulation associated with ventricular septal defect.

### Type 4—right-sided, mirror-image V, transverse

The AD lies mainly on a transverse plane extending directly from the main PA anteriorly to the descending aorta posteriorly. The PA is located anteriorly and to the right as in a mirror-image atrial arrangement. On axial views, the AA and AD confluence resembles the shape of the letter “*V*”, but a mirror-image compared of the normal orientation. In complete situs inversus, this pattern can be confused with normal anatomy if the right and left sidedness of the fetal thorax have been not systematically assessed by the examiner.

### Type 5—right-sided, s-shaped, transverse

**Fig. 5:**
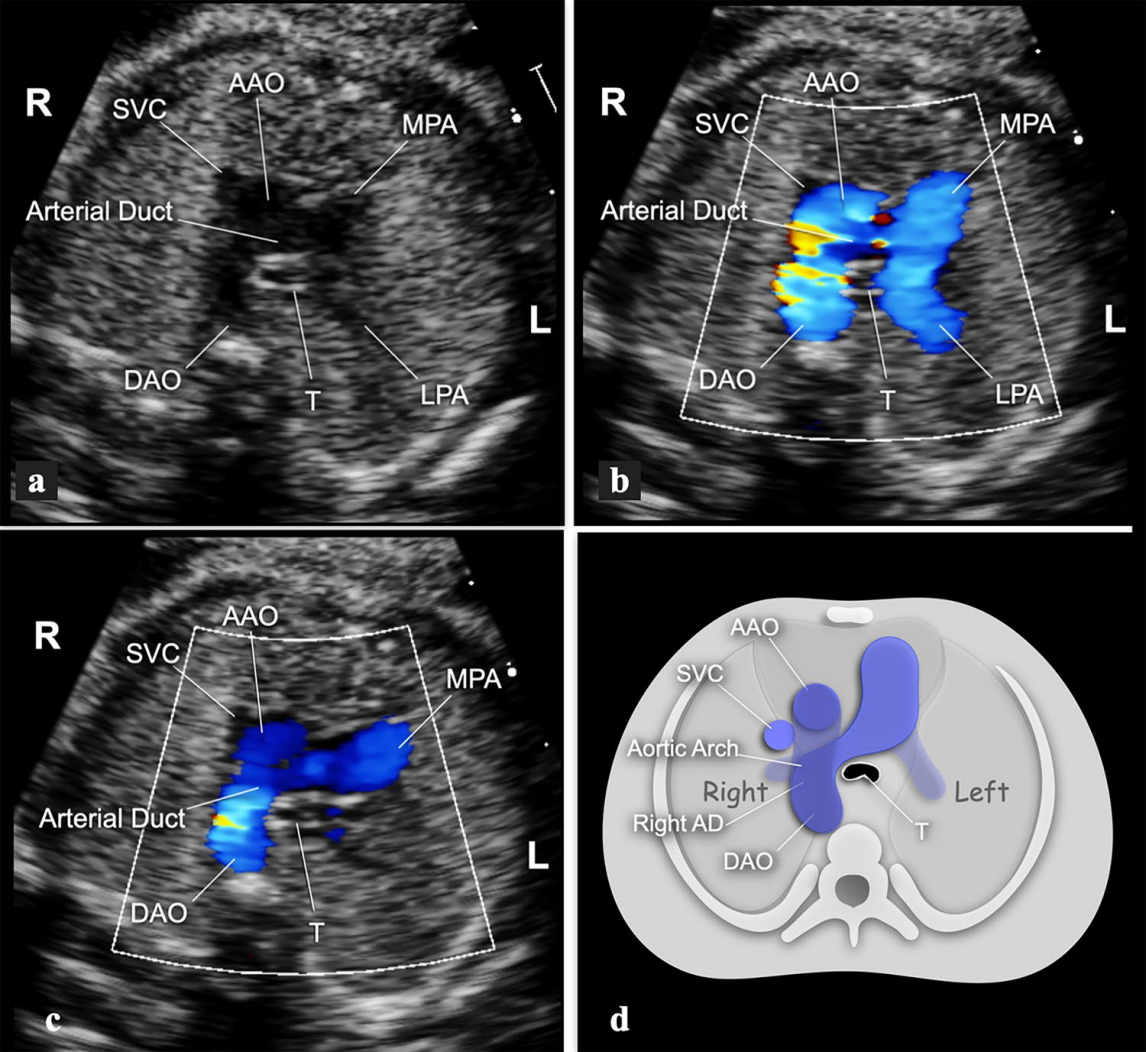
Three-vessel and trachea view of a 28-week gestational-age fetus with right AA and type 5 AD. The gray-scale image (**a**) shows how the right pulmonary artery runs anteriorly to the trachea and gives origin to a right-sided AD which joins the descending aorta at the anterior-right corner of the spine and leaves the trachea to the left. Posteriorly to the main pulmonary artery, the left pulmonary artery is seen. In contrast to the normal mirror-image “*V*” type, the corresponding color flow image resembles that of the letter “H” (**b**) or an inverted “h” (**c**) when the left pulmonary artery is not included in the section. **d** Cartoon of the same section shown in a-c. This case was not associated with intracardiac anomalies. *AAO* ascending aorta, *AD* arterial duct, *DAO* descending aorta, L left, *MPA* main pulmonary artery, *LPA* left pulmonary artery, *R* right, *SVC* superior vena cava, *T* trachea

The AD lies on a transverse plane, between the proximal right PA and the descending aorta. The first part of the AD runs to the right, anteriorly to the trachea. The second part bends posteriorly to join the descending aorta on the right-anterior corner of the spine (Fig. 5a). This view resembles a capital letter “H” (Fig. 5b) or an inverted lowercase “h” (Fig. 5c), depending on whether the left PA is included in the section plane or not. The long-axis view of the AA allows a useful cross-check in these cases. In RAA and right AD of this type, two vessels will be seen in cross section behind the ascending aorta instead of only the right PA seen in normal anatomy (Suppl. Fig. IIa, b).

### Type 6—bilateral

In these cases, two ADs are present, one on each side of the trachea. Although different combinations are possible, our series included two identical cases where a type 5 and a type 2 AD were associated with disconnection of the left PA (Suppl. Fig. IIIa-d). In such cases, both type 2 and type 5 ultrasound characteristics of the AD will be present in the same patient.

### Type 7—absent or unidentifiable

This was a diagnosis of exclusion. In this group, we included cases in which the AD was absent or unidentifiable, or when major aortic-pulmonary collaterals were seen without a recognizable confluence of the PA fed by an AD at the usual sites. For each type of AD the incidence of associated major CHD was calculated.

Statistical analysis was performed using SPSS Statics Base 22.0 (SPSS Inc, Chicago, IL, USA) and Microsoft Office Excel (Microsoft Corp., Redmond, WA, USA). Data regarding continuous variables were expressed as number and percentages. Chi square test was used for continuous variables. A p-value < 0.05 was considered significant.

## Results

Prenatal findings are summarized in Table 1.

**Table 1:** Arterial duct types in right aortic arch with and without congenital heart disease

Ductus arteriosus type	(1) U shaped	(2) I-o shaped	(3) From undersurface of AA	(4) Mirror-image V	(5) H shaped	(6) Bilateral	(7) Absent
No. Cases	42	10	11	9	9	2	15
Relationship to trachea	To the Left	Anterior to the Left	To the Right	To the Right	Anterior to the Right	Left-sided: type 2 Right-sided: type 5	–
Course/ Location plane	From MPA to desc. AO Transverse	From BCA to PA Vertical	From AA to PA Vertical	From MPA to desc. Ao Transverse	From MPA to desc. AO Transverse	(a) From RPA to desc. AO/transverse (b) from BCA to left PA, vertical	–
Flow direction	Anterograde	Retrograde	Retrograde	Anterograde	Anterograde	Left-sided: retrograde Right-sided: anterograde	–
Viscero-atrial arrangement	Solitus	Solitus	Solitus 6 Ambiguous 5	Inversus Ambiguous	Solitus Ambiguous	Solitus	Solitus Ambiguous
Without CHD	38	0	0	1	3	2	0
With CHD	4	10	11	8	6	0	15
Types of CHD/ No. cases	VSD: 1 trabecular AVSD: 1 DORV: 1 AA Interruption: 1	TOF-PS: 5 TOF-PA: 4 VSD, DORV, PA: 1	Solitus TOF-PS: 2 TOF-PA: 3 TOF-APV: 1 Right isomerism AVSD, DORV, PS: 2 AVSD, DORV, PA: 2 AVSD, VA disc, PA: 1	Inversus Dextrocardia, AzC: 1 Dextrocardia, VSD: 2 Dextrocardia, LVOT obstr: 1 Left isomerism AzC, AVSD: 1 AzC, dextroc: 1 AzC, AVSD, DORV: 1 Right isomerism TA: 1	Solitus AA interruption 2 Left Isomerism AzC, AVSD: 1 Right isomerism AVSD, HLV, DORV: 1 AVSD, DORV: 1 L-loop, VA discordance: 1	–	Solitus TOF-PS: 3 TOF-PA: 2 TOF-APV: 6 A Trunk: 2 Right isomerism AVSD, DORV, PA: 1 Left isomerism Dextrocardia, AVSD, PA: 1

*AA* aortic arch, *AO* aorta, *APV* absent pulmonary valve, *AVSD* atrioventricular septal defect, *AzC* azygos continuation, *BCA* brachiocephalic artery, *CCTGA* congenitally corrected transposition, *CHD* congenital heart disease, *Desc.* Descending, *DORV* double outlet right ventricle, *HLV* hypoplastic left ventricle, *LVOT* left ventricular outflow tract, *MPA* pulmonary artery, *PA* pulmonary atresia, *PS* pulmonary stenosis, *TA* tricuspid atresia, *TOF* tetralogy of Fallot, *A Trunk* arterial trunk, VSD ventricular septal defect

### Type 1

Type 1 was the most frequently encountered AD type in our series and occurred in 42/98 (43%) cases. Only 4 cases were associated with CHD and 3 had extracardiac anomalies including 1 surviving case with trigonocephaly, cleft palate, and polyhydramnios, 1 case where miscarriage occurred with right multicystic kidney and imperforate anus, and 1 case in which the pregnancy was terminated with bilateral multicystic kidney. Fetal karyotype was assessed in 22/38 (58%) of cases without CHD and was normal in all patients. Out of the 36 continuing pregnancies, 7 (19%) cases were lost to follow-up.

Out of the 38 cases without associated CHD, the prenatal diagnoses of the branching patterns were: 32 cases with ALSA, 5 with mirror-image branching and 1 with DAA. In 5 cases (1 with ALSA and 4 with mirror-image branching), the prenatal diagnosis was changed postnatally to DAA with distal atresia of the left limb (Table 2).

**Table 2:** Pre- and postnatal diagnosis of branching pattern in type 1 AD not associated with CHD

	With ALSA	Mirror-image branching	DAA
Prenatal diagnosis	32	5	1 two complete limbs
Postnatal diagnosis	23*	1	1 two complete limbs 4 distal atresia of the left limb
Autoptic	1¶		1§

*AA *aortic arch, *AD* aortic duct *ALSA* aberrant left subclavian artery, *CHD* congenital heart disease, *DAA *double aortic arch

*7 lost to postnatal follow-up, 1¶ intrauterine death, 1§ termination

Symptoms attributable to a vascular anomaly appeared in 12/29 (41%) of surviving patients with complete follow-up (median age of 38.2 months, range: 0.6 day to 138 months). After neonatal discharge, 6/23 (26%) cases with definitive diagnosis of ALSA had weak or absent arterial pulses in the left upper limb at their first outpatient check. This finding was not accompanied by other signs or symptoms. On CT scan, we found significant stenosis of the proximal portion of the ALSA (Suppl. Fig. IVa) in 4 patients. Of these, 3 underwent surgical repair with left subclavian artery reimplantation and resection of the arterial ligament.

Out of 6 cases with a definitive diagnosis of DAA, 1 case underwent termination while 5 developed symptoms of airway or esophageal obstruction and had surgical repair at the mean age of 8.1 months (range 2.5 to 19).

In the majority of cases (38/42; 90%), the finding of U-shaped AD was isolated, not associated with major CHD (p < 0.001).

### Type 2

Type 2 AD was observed in 10 cases with RAA (10%). Tetralogy of Fallot (TOF) was present in 9 cases, 5 with stenosis, and 4 with atresia of the pulmonary valve. One case had a TOF-like double outlet right ventricle with pulmonary valve atresia. Therefore an association with major CHD occurred in all cases of type 2 AD (p = 0.0026).

### Type 3

There were 11 cases of type 3 AD in our series (11%). All fetuses had CHD with severe pulmonary outflow obstruction (POO) and duct-dependent pulmonary circulation. Therefore an association with major CHD occurred in all cases of type 3 AD (p = 0.0045).

### Type 4

There were 9 cases (9%) with type 4 AD including 5 cases with situs inversus and 4 cases with isomerism of the atrial appendages. In all but 1 case with situs inversus, CHD was present in variable forms (p = 0.0325), none of which were associated with POO.

### Type 5

RAA and right AD of type 5 was observed in 9 cases (9%) including 3 cases with situs solitus, no associated CHD, and mirror-image branching of the AA. These cases were characterized by an uneventful postnatal course. Only 1 case, where partial agenesis of the corpus callosum was detected prenatally, developed mental retardation. However, in 3/3 cases, asymptomatic hypoplasia of the left PA was found on postnatal echocardiography. The other 6 cases had various forms of CHD (p = 0.4642) but none of these were associated with POO.

### Type 6

Our series include 2 cases (2%) of bilateral AD. Both cases presented both type 2 and type 5 AD associated with disconnection of the left PA from the main pulmonary trunk. There were no associated intracardiac anomalies (p = 0.1134). In the first historical case, the disconnected PA was identified only postnatally, when reconnection of pulmonary arteries was no longer possible [13]. In the second, prenatal diagnosis was complete and a timely surgical reconnection of the left PA was accomplished in the first weeks of life. This patient has partial duplication of chromosome 9 and Y, and is apparently asymptomatic at 10 years of age [14].

### Type 7

The AD was absent or not identifiable in 15/98 (15%) cases with RAA. Of these, 13 had situs solitus, and there was 1 case each of right and left isomerism. All 15 cases had associated CHD (p = 0.001). Besides the well-known association between TOF and absent pulmonary valve (6 cases), 5 cases had classic TOF, 3 with stenosis, and 2 with atresia of the pulmonary valve. AD was also missing in 2 cases with heterotaxia, both associated with complex CHD and pulmonary atresia.

## Discussion

In our series, RAA with left AD and ALSA (type 1a) occurred most frequently. Despite potential vascular ring, 22/23 (96%) type 1a patients developed no esophageal or airway obstruction signs during follow-up over a mean 38.2 months. However, in 6 (26%) cases, weak/absent arterial pulses in the left upper limb were found within the first postnatal months. Prenatally detected proximal stenosis of ALSA has been reported in one case initially [15], then in a series of 10 [16]. Easily missed in early years, this may remain asymptomatic for years then later cause subclavian steal or severe vascular complications [17, 18]. Transcatheter balloon angioplasty, a potential alternative to surgery, is neither definitive nor eliminates the contribution of the arterial ligament to potential formation of vascular ring. Therefore, we think surgical resection of the arterial ligament and reimplantation of the ALSA is a superior treatment.

RAA with left AD and mirror image branching (type 1b) is considered rare, usually manifesting complete vascular ring and early symptoms [19]. Herein, 1/6 type 1b patients had esophageal obstruction within the first month postnatally, and 4/6 required surgery at mean 9.5 months due to airway obstruction symptoms. Despite belief in easy prenatal diagnosis of classic DAA, terminal portions of the left arch can be atretic. This is difficult to distinguish from RAA with mirror-image branching pattern (Suppl. Fig. IVb, c). Our unusually high proportion of these cases explains the high rate of prenatal misclassification between type 1b and 1c, as similarly recently reported [12, 20, 21]. Published criteria are useful for making these distinctions postnatally [22]. We consider RAA with mirror image branching and type 1 AD equivalent to DAA, since both share similar clinical characteristics postnatally.

CHD is common with type 2 AD (90–100%, p < 0.01), most often TOF with stenosis or atresia of the pulmonary valve [23]. All cases in our series had TOF or equivalents associated with stenosis or atresia of pulmonary valve. Although rare, this can affect the second duct in bilateral AD with disconnection of pulmonary arteries.

We observed type 4 AD in situs inversus or left isomerism. Without associated CHD, it can go undetected without systematic evaluation of fetal thorax sidedness. Despite missed abnormal situs, some cases were referred for evaluation because of associated CHD. In situs inversus, the AA and AD are both on the right and no vascular ring forms. In situs inversus, AA and AD anomalies may occur similarly to in situs solitus. Consequently, evaluation must involve similar criteria. One of our cases with ALSA even remained asymptomatic in a similar way to an isolated aberrant right subclavian artery in left AA and situs solitus.

Recent studies described prenatal diagnosis of RAA and right “H” shaped AD (type 5) [24–26]. All except one case with double outlet right ventricle had normal intracardiac anatomy and confluent pulmonary arteries, and were benign with no vascular ring. Some authors [26, 27] emphasized how this can be overlooked if examiners miss that both arches are right of the trachea, and because the V-sign is maintained. In our experience, different ultrasound characteristics make it difficult to confuse with the mirror-image type. In our series, 3 cases with situs solitus and no associated CHD had smooth postnatal course, confirming the condition’s benignity. However, postnatal assessment revealed asymptomatic hypoplasia of left pulmonary artery in all cases. This association has been reported in a postnatal series [23]. Of note, with this AD type, postnatal complications may occur that require specific therapeutic approaches above those needed with normally-positioned AD [28–31].

Disconnection of pulmonary arteries with bilateral AD is rare, occurring more frequently with CHD, particularly TOF. Without CHD, a right AA and bilateral AD is common. Disconnection always occurs on the opposite side of the AA [32]. Postnatally, AD closure interrupts flow to the duct-dependent branch of PA, causing proximal thrombosis, and loss of ipsilateral lung for gas exchanges. Previously, this erroneously led to belief that the disconnected PA was absent. This malformation is also difficult to diagnose postnatally. Besides transthoracic echocardiography, CT, MRI and ventilation perfusion lung scanning are useful. Transseptal cardiac catheterization for retrograde pulmonary vein wedge angiography is often necessary to visualize the disconnected PA. Long-term complications include recurrent pneumonia, fatigue, hemoptysis and pulmonary hypertension [33–37].

Diagnosing nonconfluent pulmonary arteries prenatally is difficult. If missed, irreversible loss of the left lung to gas exchange may result [13]. Correct prenatal diagnosis allows tertiary center delivery planning and maintenance of left AD and PA patency with prostaglandin E1 for AD stenting and or surgical reconstruction of PA confluence [14, 38]. Particularly with right-sided AA and AD, normal confluence of the pulmonary arteries must always be assessed, and disconnection of one artery discounted.

In severe POO with ventricular septal defect, the origin, course and size of AD are abnormal, and flow is usually retrograde [39]. In pulmonary atresia with ventricular septal defect for example, the AD often arises with an acute angle from the undersurface of the AA and in TOF may be absent or arise from a subclavian artery [40–42]. The appearance makes it difficult to identify on fetal echocardiography. Investigation into unusual AD placement in non-standard planes is required before concluding absence. As expected, type 2, 3, and 7 were always associated with CHD (p < 0.01) and moderate-to-severe POO. Even though type 2 and 3 AD lies on a vertical fetal plane, we believe that with reasonable reliability, its position can be suspected using axial projections of the fetal thorax confirmed with sagittal views. In conclusion we believe that the characterization by ultrasound of the specific typology of AD, according to the anatomo-sonographic characteristics listed above, allow a better definition of the diagnosis and can offer useful prognostic elements.

### Limitations

The major study limitation is the retrospective nature. Moreover, most examinations were performed because the gynecologist suspected RAA or DAA. Therefore, due to referral bias, the results are not applicable to the general population. Imaging techniques and the examiners’ experience improved with time. The very low percentage of chromosomal and extracardiac anomalies in our series probably reflect selection bias, since not all fetuses with these anomalies are referred to cardiologists. Because of religious and cultural preferences, a limited number of chromosomal analyses were performed. This could explain the low incidence of chromosomal abnormalities. Several patients relocated, which explains the 19% of patients lost to follow-up. Consequently, postnatal findings may be under-represented.

## Supplementary Information

Below is the link to the electronic supplementary material.

Supplementary file1 (DOC 8 kb)

Supplementary file2 (TIF 3814 kb)

Supplementary file3 (TIF 1422 kb)

Supplementary file4 (TIF 2225 kb)

Supplementary file5 (TIF 3188 kb)

## Data Availability

The data that support the findings of this study are available on request from the corresponding author. The data are not publicly available due to privacy or ethical restrictions.
