# Supplementary figures and images for: The multiform sonographic spectrum of arterial duct in right aortic arch
Source: Int J Cardiovasc Imaging. 2021 Jul 8;37(12):3385–95. doi: 10.1007/s10554-021-02325-w (PMC8604842; doi:10.1007/s10554-021-02325-w)

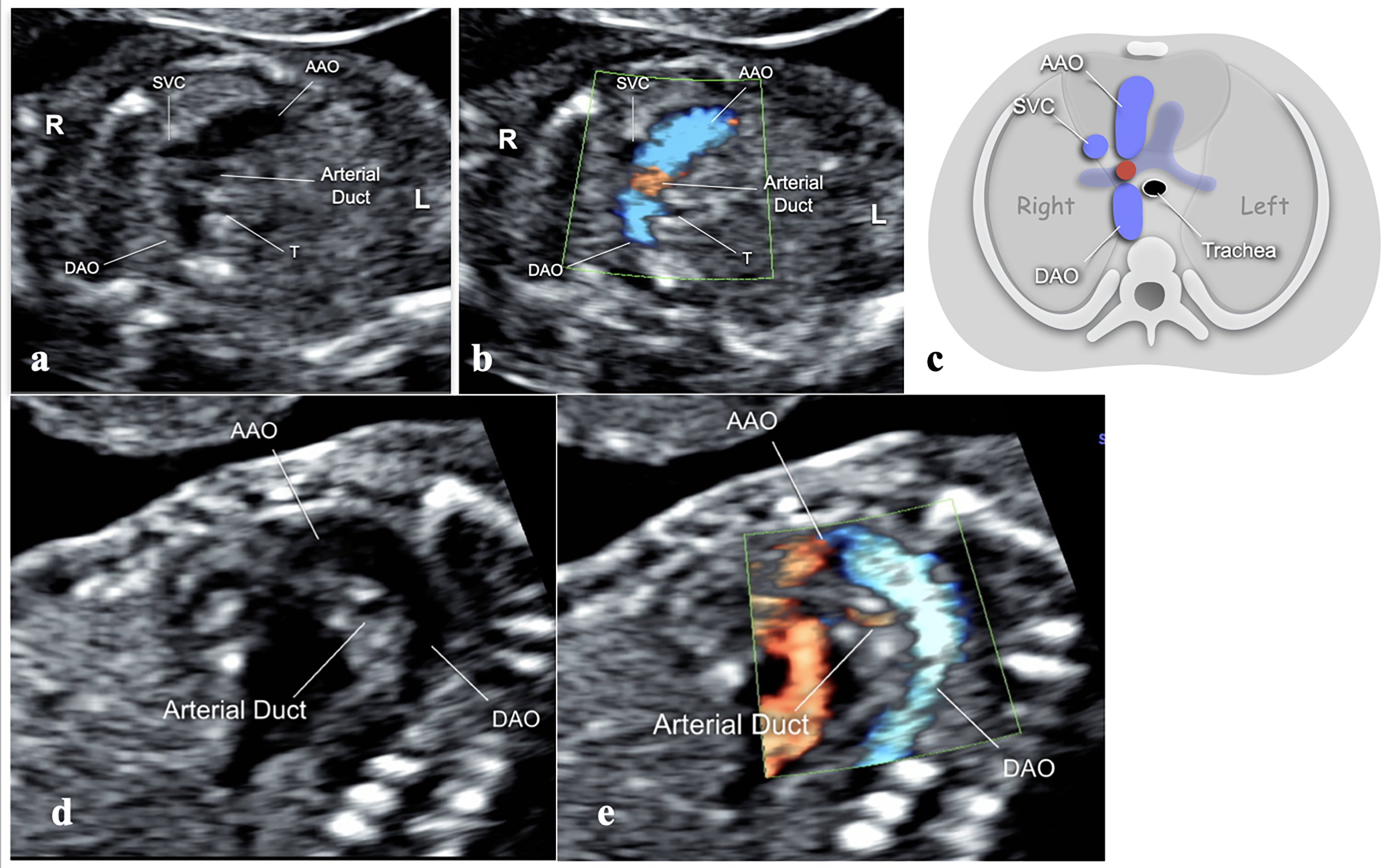

Supplement: Supplementary file 2 — Supplementary file2 (TIF 3814 kb) [file 10554_2021_2325_MOESM2_ESM.tif]

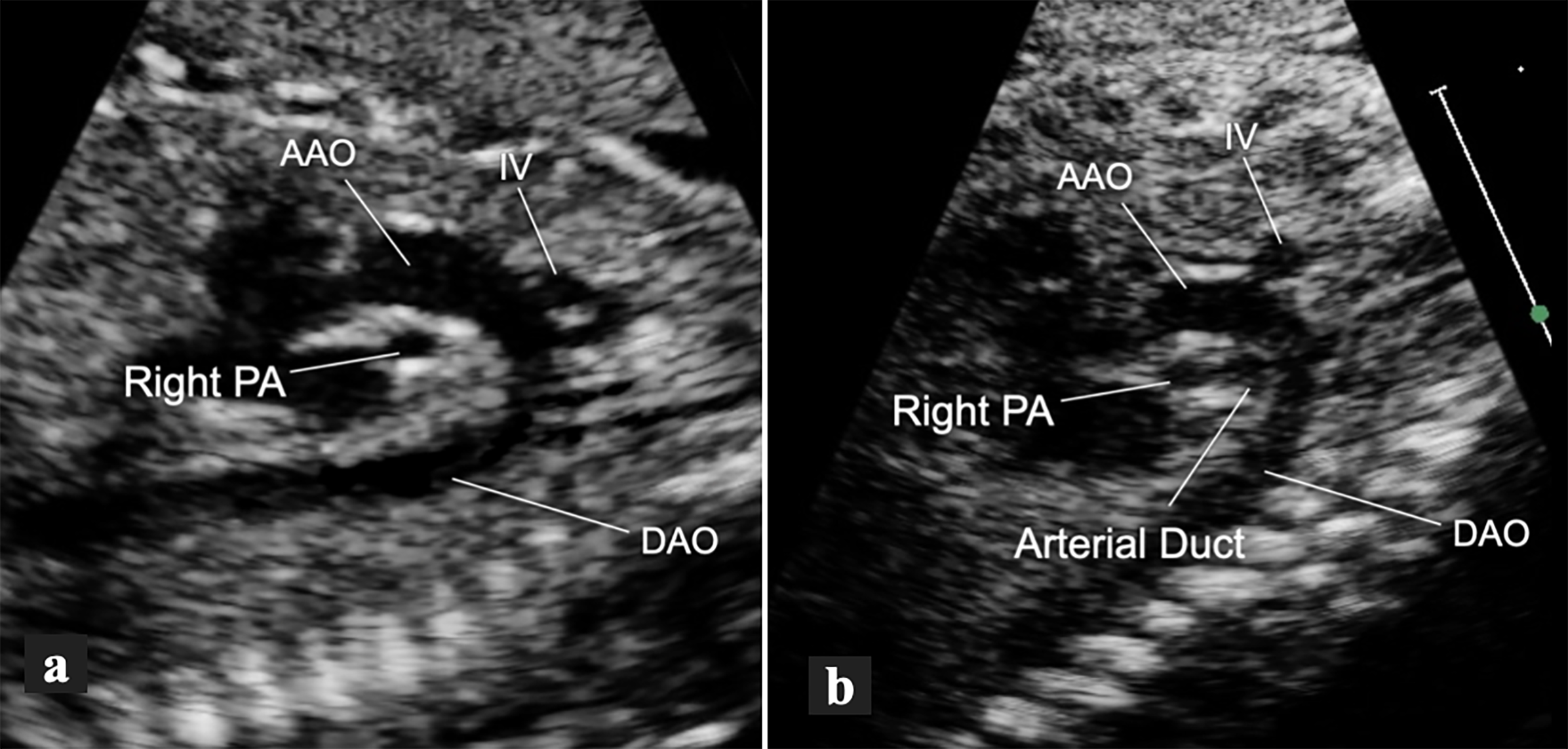

Supplement: Supplementary file 3 — Supplementary file3 (TIF 1422 kb) [file 10554_2021_2325_MOESM3_ESM.tif]

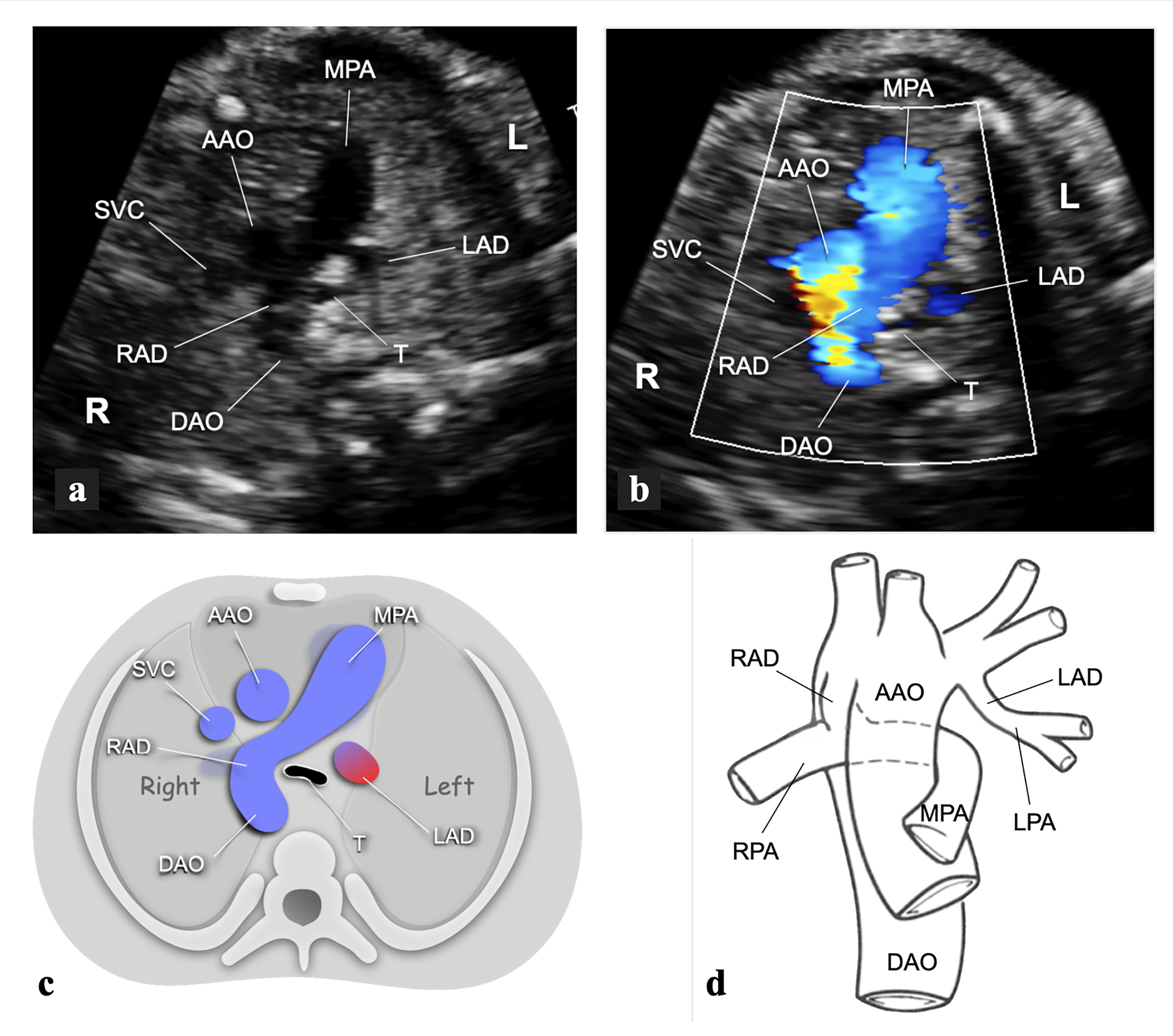

Supplement: Supplementary file 4 — Supplementary file4 (TIF 2225 kb) [file 10554_2021_2325_MOESM4_ESM.tif]

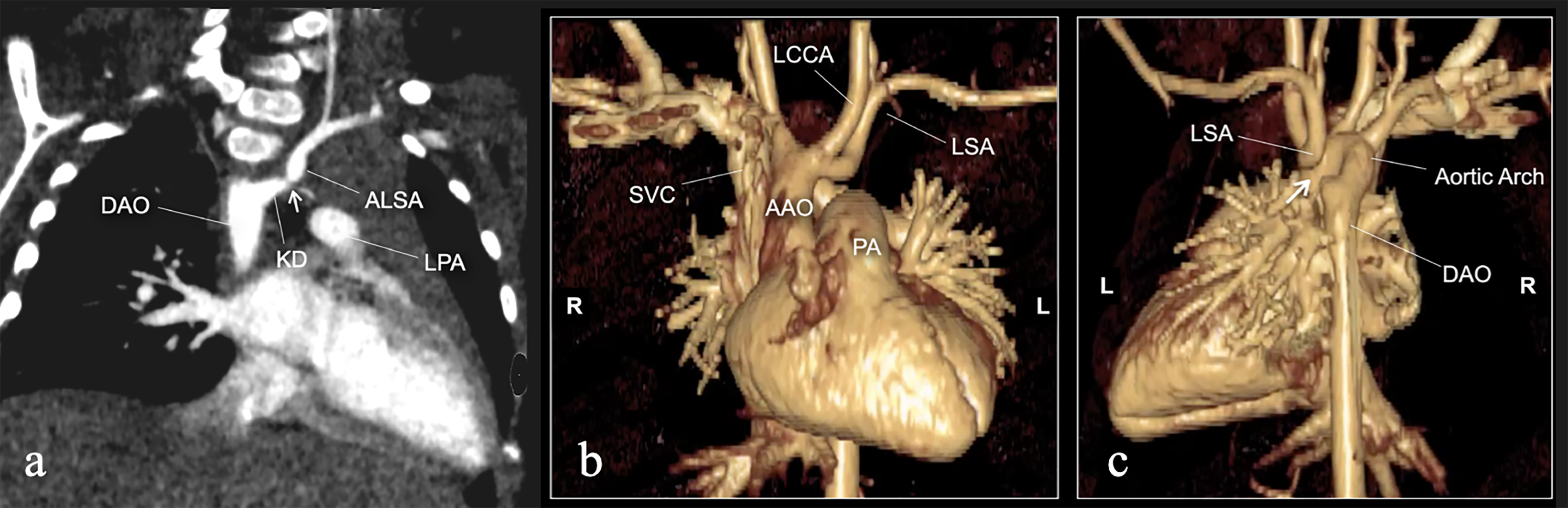

Supplement: Supplementary file 5 — Supplementary file5 (TIF 3188 kb) [file 10554_2021_2325_MOESM5_ESM.tif]
