# Supplementary material for: The multiform sonographic spectrum of arterial duct in right aortic arch
Source: Int J Cardiovasc Imaging. 2021 Jul 8;37(12):3385–95. doi: 10.1007/s10554-021-02325-w (PMC8604842; doi:10.1007/s10554-021-02325-w)
Supplement: Supplementary file 3 [file 10554_2021_2325_MOESM1_ESM.doc]

Suppl. Fig. I. Gray-scale (a), color flow mapping image (b) and the corresponding cartoon (c) of the transverse views of a 16-week gestational-age fetus with RAA and AD type 3. This fetus had right isomerism, malposition of the great arteries with atresia of the pulmonary valve. The axial projections show an oblique section of the proximal and distal aortic arch with a transverse section of the AD in between. Gray-scale (d) and color flow mapping image (e) of the long-axis views of the aortic arch in the same patient. These pictures show how the AD originates from the concavity of the AA to run inferiorly, with a vertical course, and join the confluence of the pulmonary artery.

AAO ascending aorta, DAO descending aorta, L left, R right, SVC superior vena cava, T trachea.

Suppl. Fig. II. Long-axis view of the aortic arch in a normal fetus (a) and in a case with RAA and right AD type 5 (b). In normal anatomy, the cross section of the right pulmonary artery only is displayed posteriorly to the ascending aorta. In type 5 AD, two vessels are displayed in cross section: the right pulmonary artery inferiorly, and the arterial duct superiorly.

 AAO ascending aorta, DAO descending aorta, IV innominate vein, PA pulmonary artery.

Suppl. Fig. III. Gray-scale (a), color flow image (b) and schematic cartoon (c) of three-vessel and trachea view in a 25-week gestational-age fetus with right AA and bilateral arterial duct (Type 6). In this case, two ADs are present: the right-sided AD is type 5 and the left-sided AD is type 3. This latter perfuses at full channel the left pulmonary artery, which is disconnected from the main pulmonary artery. The images show the AA and the AD to the right of the trachea and the disconnection of the left pulmonary artery from the main trunk. d) Schematic representation of this vascular anomaly.

 AAO ascending aorta, DAO descending aorta, L left, LAD left arterial duct, LPA left pulmonary artery, MPA main pulmonary artery, R right, RAD right arterial duct, RPA right pulmonary artery, SVC superior vena cava.

Suppl. Fig. IV. Angio-CT in right-anterior oblique projection in an infant with RAA and aberrant left subclavian artery (a). The image highlights the distal origin of the left subclavian artery from the first tract of the descending aorta and shows significant stenosis at its origin (white arrow). Multislice CT rendering (b,c) at 15-month infant of our series with symptoms of persistent respiratory obstruction following an upper airway infection. This case was referred to fetal echocardiography for suspect double aortic arch but subsequently reclassified as DAA with distal atresia of the left limb. The anteroposterior view (b) shows the right-sided aortic arch, and the right-sided aortic arch  the left-sided brachiocephalic artery from which the left common carotid artery and left subclavian artery originate. The postero-anterior view (c) shows a left-sided descending aorta with a small gap (white arrow) between the left subclavian artery and the aortic stump, characteristic features of double aortic arch with a small atretic segment.

 AAO ascending aorta, ALSA aberrant left subclavian artery, DAO descending aorta, KD Kommerell's diverticulum, L left,  LCCA left common carotid artery, LPA left pulmonary artery, LSA left subclavian artery, PA pulmonary artery, R right, SVC superior vena cava.
